# Tumor Blood Flow Is a Predictor of Radiotherapy Response in Patients With Nasopharyngeal Carcinoma

**DOI:** 10.3389/fonc.2021.567954

**Published:** 2021-08-06

**Authors:** Xiujuan Cao, Jian Song, Juan Xu, Guanzhong Gong, Xinhua Yang, Ya Su, Lizhen Wang, Xiaodong Bai, Man Hu, Yong Yin

**Affiliations:** ^1^Department of Radiation Oncology, Shandong Cancer Hospital, Cheeloo College of Medicine, Shandong University, Jinan, China; ^2^Department of Radiation Oncology, Shandong Cancer Hospital and Institute, Shandong First Medical University and Shandong Academy of Medical Sciences, Jinan, China; ^3^Medical Imageology, Shandong Medical College, Jinan, China; ^4^Department of Radiation Oncology Physics and Technology, Shandong Cancer Hospital and Institute, Shandong First Medical University and Shandong Academy of Medical Sciences, Jinan, China; ^5^Department of Plastic Surgery, Southern University of Science and Technology Hospital, Shenzhen, China

**Keywords:** tumor blood flow, radiotherapy, nasopharyngeal carcinoma, predictor, response

## Abstract

**Purpose:**

The aim of this study was to evaluate tumor blood flow (TBF) as a predictor of radiotherapy response for nasopharyngeal carcinoma (NPC).

**Materials and Method:**

A total of 134 patients were divided into two groups, the complete response (CR) group and the partial response (PR) group based on RECIST 1.1 recommendations. The statistical difference was evaluated for pre- and mid- or post-treatment TBF and changes of TBF for tumors and metastatic lymph nodes between CR and PR, respectively. The receiver operation characteristic (ROC) curve was utilized to evaluate the accuracy of TBF in predicting the response of radiation therapy. The association between TBF and SUVmax was also investigated.

**Results:**

The reduction of TBF in CR was significantly lower than that in PR for primary tumors (*P <*0.001) and metastatic lymph nodes (*P <*0.001). The multivariate logistic regression analysis indicated that the reduction of TBF is an independent predictor of the response of radiation therapy for primary tumors (*P <*0.001) and metastatic lymph nodes (*P <*0.001). The accuracy of TBF reduction in predicting the response of radiation therapy was 0.817 in primary tumors and 0.924 in metastatic lymph nodes, respectively. No significant correlation was observed between the TBF values and SUVmax of primary tumors (*r* = -0.008, *P* = 0.954) and metastasis lymph nodes (*r* = -0.061, *P* = 0.652).

**Conclusion:**

This study suggests that the reduction of TBF is a promising parameter for evaluating the response of radiation therapy.

## Introduction

Nasopharyngeal carcinoma (NPC) is one of the distinctly distributional cancers and is geographically prevalent in southeast Asia and southern China (1). It is well known that Epstein-Barr virus (EBV) infection, host genetics, and environmental factors contribute to the occurrence of NPC, and EBV DNA testing is used to detect, prognose, and assess tumor response earlier (2, 3). The pre-treatment EBV DNA load was correlated positively with progression of NPC after curative treatment, plasma EBV DNA immediately post treatment had the potential as a quantitative biomarker of tumor response assessment to guide the use of aggressive adjuvant chemotherapy (4). There is no abundant assay with robust analytical characteristics for clinical utility to inform treatment management. Based on monitoring treatment response, it is therefore essential to extract reliable prediction factors that could divide patients into low- or high-risk groups. The low-risk group may avoid ineffective therapies and prevent unnecessary adverse effects, while the high-risk group may benefit from aggressive therapies.

A reliable and accurate predictor of early response can improve patient care by tailoring treatment and optimizing follow-up plans. The Response Evaluation Criteria in Solid Tumors (RECIST 1.1) criteria is usually recommended to assess the morphologic changes of the tumor with computed tomography (CT) or magnetic resonance imaging (MRI) (5). Fujima reported that anatomic imaging changes had several limitations in predicting treatment response (6) and were not sufficient for detecting the intratumoral heterogeneity. ^18^F-Fluorodeoxyglucose positron emission tomography (^18^F-FDG PET) is another technique used to evaluate the recurrence, metastasis, or prognosis of NPC, by several semi-quantitative parameters, such as standardized uptake value (SUV), volumetric parameters: metabolic tumor volume (MTV), and total lesion glycolysis (TLG) (7). However, PET-CT has low spatial resolution and a high false-positive rate, so these parameters cannot insufficiency predict treatment response to radiotherapy (8). Shi et al. compared ^18^F-FLT and ^18^F-FDG PET/CT in monitoring and predicting tumor regression of NPC; parameters of FDG PET were more strongly correlated to treatment response than those of FLT PET (9). Hanamoto et al. explored whether pre-treatment metabolic tumor volume (MTV) and total lesion glycolysis (TLG) of PET-CT can predict the local response of laryngohypopharyngeal cancer by chemoradiotherapy (10). Xie et al. showed that SUVmax of PET-CT may be a valuable tool to predict prognosis in locally advanced NPC (11). Liu et al. suggested that the Hopkins criteria was a reliable predictive and prognostic indicator in post-treatment assessment, the addition of EBV DNA and PET/CT did not improve evaluative accuracy of therapy response (12).

Functional MRI may predict response and allow for the modification of a treatment schedule before or early in the course of treatment. Liu et al. explored whether quantitative image parameters based on contrast-enhanced MRI served as new predictive tools for NPC response to chemoradiotherapy (13). Huang et al. demonstrated that Kmean-post values were the most powerful predictor for the early treatment evaluation of NPC in the investigation of DKI and DWI (14). Tumor blood flow (TBF) can be used to determine the tumor perfusion. Previous studies suggested that TBF might lead to tumor cells becoming resistant to treatment; and several factors, such as tumor vascularity, permeability, and oxygenation, are involved in this effect of TBF (15). However, TBF is usually obtained by contrast-enhanced MRI, which may increase the risk of allergy and nephrogenic systemic fibrosis. Recently, it was reported that TBF can be achieved by the noninvasive arterial spin labeling (ASL) MRI technique without contrast agents (16). Wu et al. compared the TBF obtained from ASL-MRI and dynamic contrast-enhanced (DCE)-MRI and observed that both test methods were highly consistent (17). In this study, we explored the feasibility of TBF with ASL-MRI in quantitatively evaluating the tumor response of NPC.

## Materials and Methods

### Patients

The retrospective study protocol was approved (No. 201807036) by our clinical research ethic committee at the Cancer Institute & Hospital, ** Academy of Medical Sciences. From September 2018 to December 2019, 134 newly diagnosed nasopharyngeal carcinoma (NPC) patients were enrolled in our study and classified as stage I (n=5), II (n=20), III (n=75), and IV (n=34) patients according to the 8th edition of the American Joint Committee on Cancer staging manual with the following inclusion criteria: (1) Age > 18 years old, ECOG ≤ 2; (2) a clear pathological diagnosis; (3) without distant metastasis; and (4) without any anti-tumor treatment before the MR examination. The exclusion criteria was: (1) Cases of previous or concurrent malignancy; (2) cases with contraindication of MRI examination, such as individuals with pacemakers, non-detachable metal objects, or claustrophobic disorder; and (3) patients without complete treatment. All the patients received definitive radiotherapy and platinum-based concurrent chemoradiotherapy. The details of standard treatment were as follows: dosing lists of cisplatin were 40 mg/m² per week or 80–100 mg/m² every 3 weeks, which is commonly acceptable as the first choice with concurrent radiation therapy. The curative radiation dose needs to reach a total of 66-70 Gy in 33-35 fractions. Two MRI examinations including ASL were obtained from each patient as follows: (1) pre-treatment, 0-5 days between the first MRI examination and the start of treatment; (2) mid-treatment, 0-1 days after receiving 50 Gy radiotherapy or post-treatment, 0-3 days from the end of radiotherapy. Owing to high costs and unavailability of medical insurance reimbursement, ^18^F-FDG PET/CT scans were only acquired from 62 patients before radiotherapy who needed to identify suspicious lesions or exclude distant metastases. Besides, fasting blood glucose concentration had to be under the level of 10.0 mmol/L for the PET/CT scan in patients with diabetes.

### MR Imaging Protocol

MR images were acquired using a 3.0 T positioning MR system (Discovery MR750, GE Medical Systems, Milwaukee, Wisconsin, USA) with a 6-channel neurovascular coil. The following sequences were employed: the axial T1WI (fast spin echo, FSE, TR= 670 ms, TE= 13.63 ms); the arterial spin labeling sequence, which is a 3D fast spin echo (FSE) spiral-based pseudo-continuous pCASL sequence (NEX= 3, bandwidth= 62.50 kHz, thickness= 3 mm, slice gap= 0 mm, FOV= 26 cm, TE= 11.4 ms, PLD 2025 ms [2.0 s]: TR/TA= 5481 ms/318 s); the axial T2WI with periodically rotated overlapping ParallEL lines with enhanced reconstruction (Propeller) (Fast Recovery FSE, TR= 7059 ms, TE= 75 ms, NEX= 1.8, bandwidth= 83.33 kHz, thickness= 3 mm, slice gap= 0 mm, FOV = 28 cm, matrix = 384, TA = 419 s), which are non-enhanced series. In addition, a contrast-enhanced scan based the axial T1WI was also accomplished by using 3D liver acquisition with volume acceleration-flexible (LAVA-Flex) with Gadolinium -DTPA -BMA (Ominscan, GE lifeScience, China) (dose 0.2 ml/kg and rate of 2.0 ml/s. The parameters were TR= 6.8 ms, TE= 2.86 ms, NEX= 1, bandwidth= 142.86 kHz, thickness= 3 mm, slice gap= 0mm, FOV= 34 cm, matrix= 296× 296, TA= 64 sec). The scan range included nasopharyngeal tumor and neck lymph node regions. In order to minimize motions during scanning, a head, neck, and shoulder thermoplastic mask was used and patients were trained to avoid moving their tongues, swallowing, or speaking as best as they could.

Data analysis was achieved on Advantage Workstation (GE Healthcare, Milwaukee, WI, USA). The following equation was used to calculate the TBF values (18):

$$BF=\frac{6000\cdot \text{λ}\cdot (\text{S}{\text{I}}_{\text{control}}-\text{S}{\text{I}}_{\text{label}})\cdot {\text{e}}^{\frac{\text{PLD}}{{\text{T}}_{1,\text{blood}}}}}{2\cdot \text{α}\cdot {\text{T}}_{1,\text{blood}}\cdot \text{S}{\text{I}}_{\text{PD}}\cdot (1-{\text{e}}^{-\frac{\tau }{{\text{T}}_{1,\text{blood}}}})}[mL/100g/\text{min}]$$

where BF is blood flow, λ represents the blood partition coefficient in ml/g, SI _control_ and SI _label_ are the time averaged signal intensities of the control and label images, respectively, and PLD is the post-labeling delay time. T_1, blood_ is the longitudinal relaxation time of blood in seconds, α is the labeling efficiency for pCASL, SI_PD_ is the signal intensity of a proton density-weighted image, and τ is the pCASL label duration. 6000 is a customary element, which changes the unit from ml/g/s to a commonly used unit, i.e., mL/100g/min.

### ^18^F-FDG PET Imaging Analysis

All patients needed to fast for at least 8 h and measure their blood glucose level before scanning. ^18^F-FDG PET/CT scans were performed 60 min after an injection of 5.55–7.40 MBq/kg of ^18^F-FDG (GE Discovery LS PET/CT). CT images were acquired on the same scanner. The PET scans were reconstructed with the CT-based attenuation correction using the ordered subset expectation maximization (OSEM) algorithm. The standard uptake value (SUV) in the region of interest (ROI) was calculated using the tissue concentration of ^18^F-FDG measured by PET/the injected FDG dose/body weight. The FDG uptake in primary tumors and metastasis lymph nodes using the maximum SUV (SUVmax) were calculated using semiquantitative analysis. If the tumor extended beyond two slices, the highest SUV value of all tumor ROIs was defined as the SUVmax.

### Tumor ROI Delineation and Evaluation

**Figure 1:**
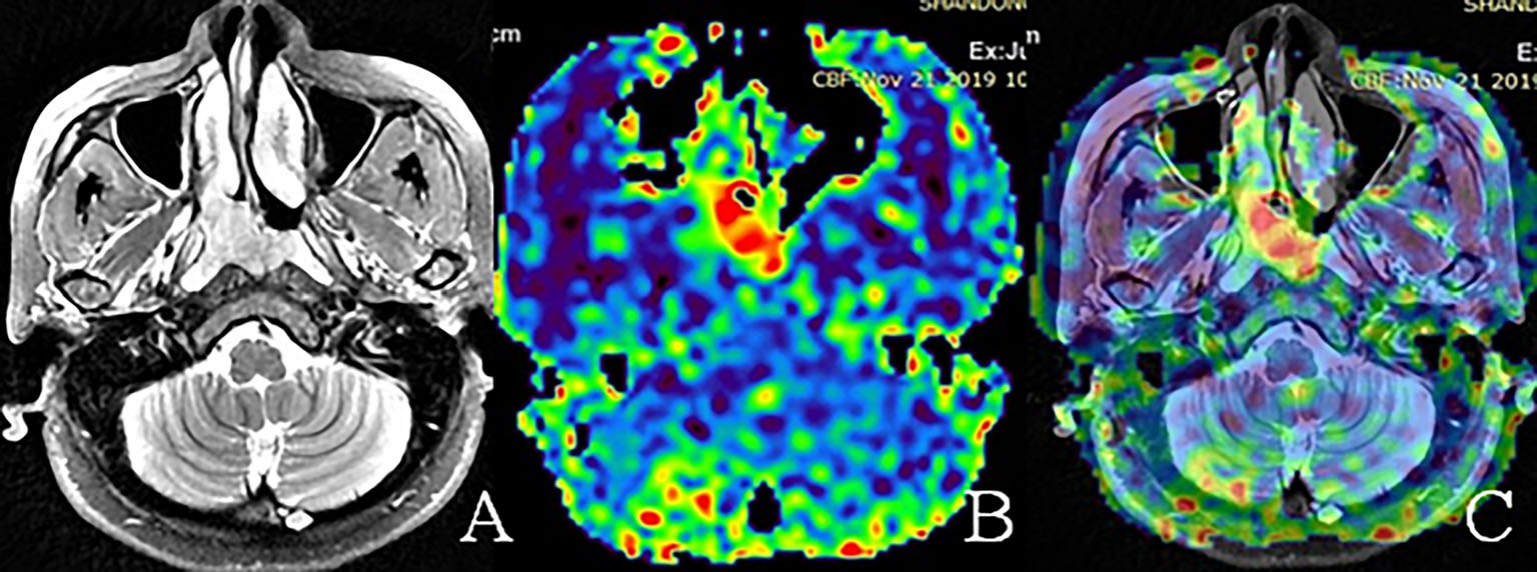
Representative examples of nasopharyngeal tumor region of interest (ROI) on different images. **(A)** ROI on the axial T2WI. **(B)** The ROI on the corresponding TBF map, which demonstrated higher perfusion compared with the surrounding tissue. **(C)** The same ROI on T2WI and ASL fusion images. The images of **(A–C)** were obtained from the pre-treatment MRI of a 66-year-old woman with a T_3_ non-keratinizing undifferentiated tumor.

The primary tumors and metastasis lymph nodes were delineated by a board-certified head and neck tumor radiologist with 10 years experience. Firstly, we delineated the polygonal ROIs along the tumor boundary for both primary tumors and all metastasis lymph nodes on axial T2WI. The skull base bone was excluded. Bone lesions are unmeasurable, while the peripheral soft tissue components can be evaluated by CT or MRI in RECIST 1.1. The diagnostic criteria for metastatic lymph nodes (LNs) were: retropharyngeal LNs > 5 mm or cervical LNs > 10 mm in shortest diameter; three or more contiguous and confluent LNs, each with shortest diameter of 8–10 mm; LNs of any size with central necrosis or a contrast-enhanced rim; LNs of any size with extracapsular extension LNs of any size with overt FDG uptake on the FDG-PET scan; non-metastatic lymph nodes were without the above features. Secondly, the axial T2WI and TBF maps derived from ASL of the same level were rigidly registered and the ROIs were propagated to the corresponding TBF map (**Figure 1**). The cystic necrosis and vessel signal void were excluded from the TBF measurement to avoid inaccurate perfusion information in the ROIs. If the ROIs were expanded for two or more slices on the TBF maps, the mean TBF values of primary tumors and all metastasis lymph nodes were calculated in each patient. The reduction rate of TBF pre- and mid- or post-treatment was calculated and evaluated for each ROI. It was calculated as follows: percentage change of TBF = (mid- or post-treatment TBF - pre-treatment TBF)/(pre-treatment TBF) × 100%.

In addition, the tumor volume (TV) was measured on contoured ROIs using the Varian Eclipse Treatment System (version 15.5, Varian Medical System, Palo Alto, CA, USA). If tumors enlarged beyond two slices, the total of the TV was calculated for all slices (19). The evaluation time of tumor response was early post-treatment, 0-3 days from the end of radiotherapy. According to the Response Evaluation Criteria in Solid Tumors criteria, by their TV changes between pre-treatment and early post-treatment MRI examinations, 134 patients were classified into two groups: the complete response (CR) group was defined as the disappearance of all tumor lesions, the partial response (PR) group was defined as a percentage reduction of TV ≥ 30%.

### Statistical Analysis

The associations between response of radiotherapy and clinicopathologic characteristics were assessed by the chi-square test. The statistical difference between pre- and post-treatment TBF, and the change of TBF between CR and PR groups were evaluated with non-paired t-test. Univariate and multivariate logistic regression models were utilized to analyze those parameters to determine whether they have independent predictive value for treatment response. In order to perform multivariate logistic regression, the variables, which were statistically significant in univariate logistic regression, were analyzed. The detected predictive values were also assessed using receiver operating characteristic (ROC) curves and area under the curve (AUC). All statistical analysis was performed using SPSS (version 22; IBM SPSS) and Graphpad prism6.0 (Graphpad Software, San Diego, CA). *P <*0.05 was considered to indicate statistical significance.

## Results

### The Clinicopathological Characteristics of Patients

**Figure 2:**
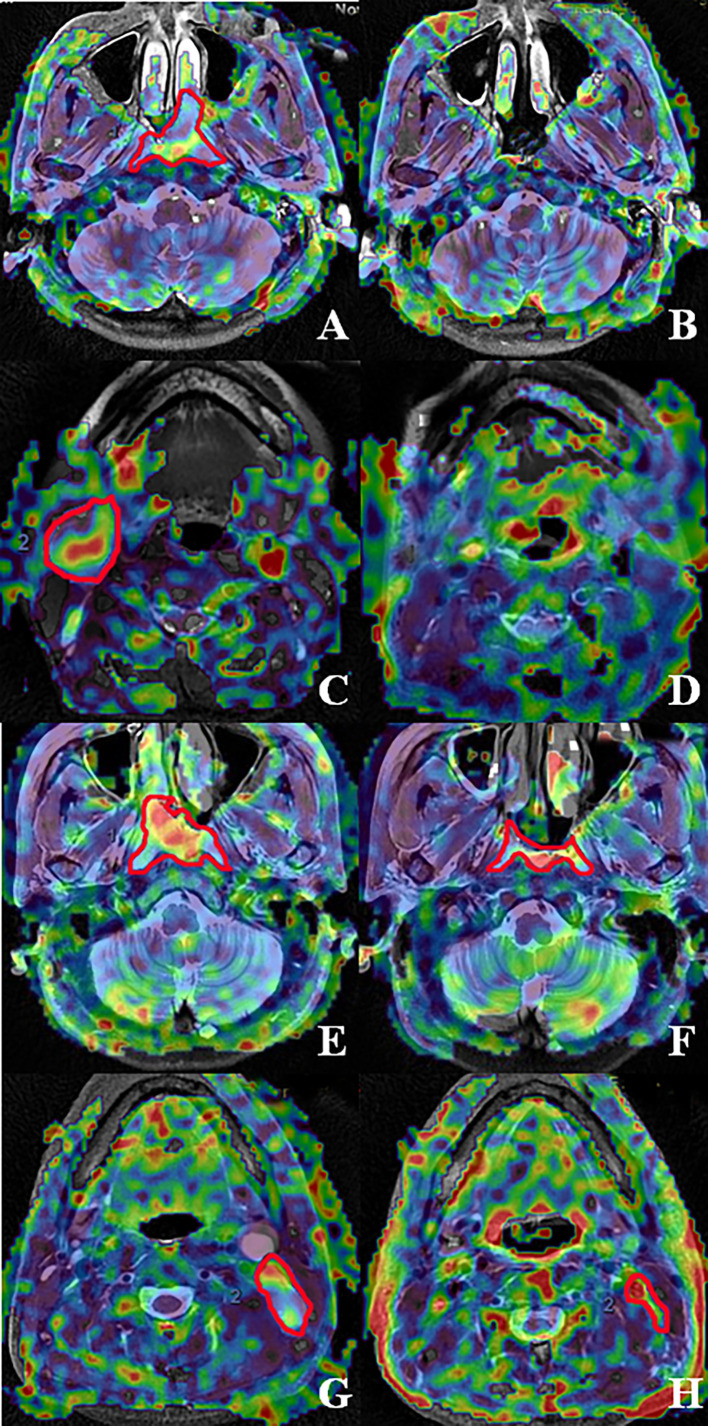
Fusion images of the ASL superimposed on T2WI acquired from patients with nasopharyngeal carcinoma accompanied by metastatic lymph nodes. The fused image by T2WI and ASL of nasopharynx lesion and metastatic lymph node pre-treatment and post-70 Gy radiotherapy. **(A–D)** represent complete response in nasopharynx lesions and metastatic lymph nodes, respectively. **(E–H)** represent partial response in nasopharynx lesions and metastatic lymph nodes, respectively. **(A, B)** The pre-and post-treatment ASL of a 51-year-old man with a T_2_ non-keratinizing undifferentiated tumor. **(C, D)** The pre-and post-treatment ASL of a 54-year-old woman with an N_3_ non-keratinizing undifferentiated tumor. **(E, F)** The pre-and post-treatment ASL of a 66-year-old woman with a T_3_ non-keratinizing undifferentiated tumor. **(G, H)** The pre-and post-treatment ASL of a 57-year-old man with an N_2_ non-keratinizing differentiated tumor.

In total, 134 patients were included in this study. In 100 patients, the TBF was evaluated when patients received 50 Gy of radiotherapy. In 34 patients, the TBF was determined when patients received 70 Gy. Notably, most of these patients (except T_1,2_N_0_) continued to receive adjuvant chemotherapy after 70 Gy. There were 120 out of 134 patients in this study with known metastatic lymph nodes. A total of 99 (73.88%) primary tumors and 46 (34.33%) metastasis lymph nodes were categorized into the CR group to radiotherapy, the remaining 35 (26.12%) and 74 (65.67%) were in the PR group (**Figure 2**). The radiotherapy response of primary tumors was significantly better than metastatic lymph nodes (*P* < 0.001). There was a significant difference in primary tumor radiotherapy response between T_1+2_ and T_3+4_ (*P* = 0.003), I+II and III+IV (*P* = 0.018). The treatment response of metastatic lymph nodes was significantly different between I+II and III+IV (*P* = 0.002). However, there was no significant correlation between treatment outcome and the other clinicopathological characteristics such as gender, age, pathology, and therapy (**Table 1**).

**Table 1:** Clinical characteristics of all patients and radiotherapy response.

Characteristic	Primary tumor		*χ* ^2^	*P-*value	LNM		*χ* ^2^	*P*-value
	CR	PR			CR	PR		
Gender								
Male	78	25	0.788	0.375	33	58	0.682	0.409
Female	21	10			13	16		
Age (years)								
≤50	48	20	0.776	0.379	21	39	0.564	0.453
>50	51	15			25	35		
Pathological type								
Keratinizing squamous	4	4	4.848	0.089	2	2	3.537	0.171
Non-keratinizing differentiated	21	3			13	11		
Non-keratinizing undifferentiated	74	28			31	61		
Tumor stage								
T1+2	63	12	9.039	0.003	23	36	0.021	0.886
T3+4	36	23			23	38		
Clinical stage								
I+II	23	2	5.575	0.018	10	2	9.405	0.002
III+IV	76	33			36	72		
Therapy								
RT alone	6	1	0.084	0.772	1	2	0.01	1.00
Concurrent CRT	93	34			45	72		

CR, complete response; PR, partial complete; LNM, lymph node metastasis; RT, radiotherapy; CRT, chemo-radiotherapy.

We measured the SUVmax of primary tumors and metastasis lymph nodes for 62 and 57 patients, respectively. The pre-treatment SUVmax were 12.85 ± 4.15 and 11.24 ± 4.35 for primary tumors and metastasis lymph nodes, respectively. The mean TBF of primary tumors and metastatic lymph nodes pre-treatment were 87.68 ± 22.36 mL/100g/min and 69.73 ± 14.73 mL/100g/min, respectively. There was no significant correlation between the TBF values and SUVmax of primary tumors (*r* = -0.008, *P* = 0.954) and metastasis lymph nodes (*r* = -0.061, *P* = 0.652).

### Associations of TBF With Radiotherapy Response

**Figure 3:**
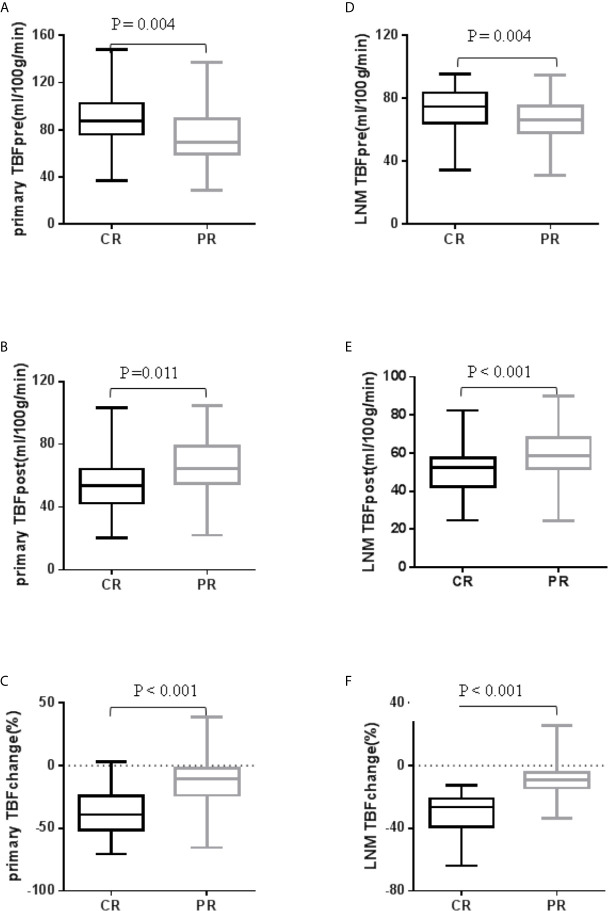
Box plot analysis of TBF between CR and PR in 134 primary tumors and 120 metastatic lymph nodes. TBF of primary tumor **(A)** before treatment and **(B)** after treatment; TBF of LNM **(C)** before treatment and **(D)** after treatment; **(E)** TBF change of primary tumor; **(F)** TBF change of LNM.

The pre-treatment TBF of patients in the PR group was significantly lower than that in the CR group, i.e., 76.56 ± 26.23 mL/100g/min *vs.* 89.43 ± 20.56 mL/100g/min in primary tumors (*P* = 0.004), and 66.31 ± 13.48 mL/100g/min *vs.* 73.87 ± 14.23 mL/100g/min in metastatic lymph nodes (*P* = 0.004). The mid- or post-treatment TBF of PR patients was significantly higher than that in CR patients, i.e., 64.78 ± 18.39 mL/100g/min *vs.* 55.79 ± 17.46 mL/100g/min in primary tumors (*P* = 0.011) and metastatic lymph nodes 59.40 ± 12.25 mL/100g/min *vs.* 50.75 ± 11.92 mL/100g/min in metastatic lymph nodes (*P* < 0.01). The reduction rate of TBF between mid- or post-treatment and pre-treatment was significantly lower in CR than those in PR (-36.49 ± 18.27% *vs.* -11.80 ± 20.74% in primary tumors, and -30.42 ± 13.17% *vs.* -9.95 ± 9.26% in metastatic lymph nodes) (*P* < 0.001 and *P <*0.001, respectively) (**Figure 3**). However, the SUVmax of primary tumors and metastatic lymph nodes in the pre-treatment period was not significantly different between CR and PR groups (12.71 ± 3.94 *vs.* 13.39 ± 5.04 in primary tumors, and 10.53 ± 4.26 *vs.* 11.76 ± 4.41 in metastatic lymph nodes, with *P* = 0.602 and *P* = 0.297, respectively).

### Independent Predictors and Accuracy Prediction of the Change of TBF

**Figure 4:**
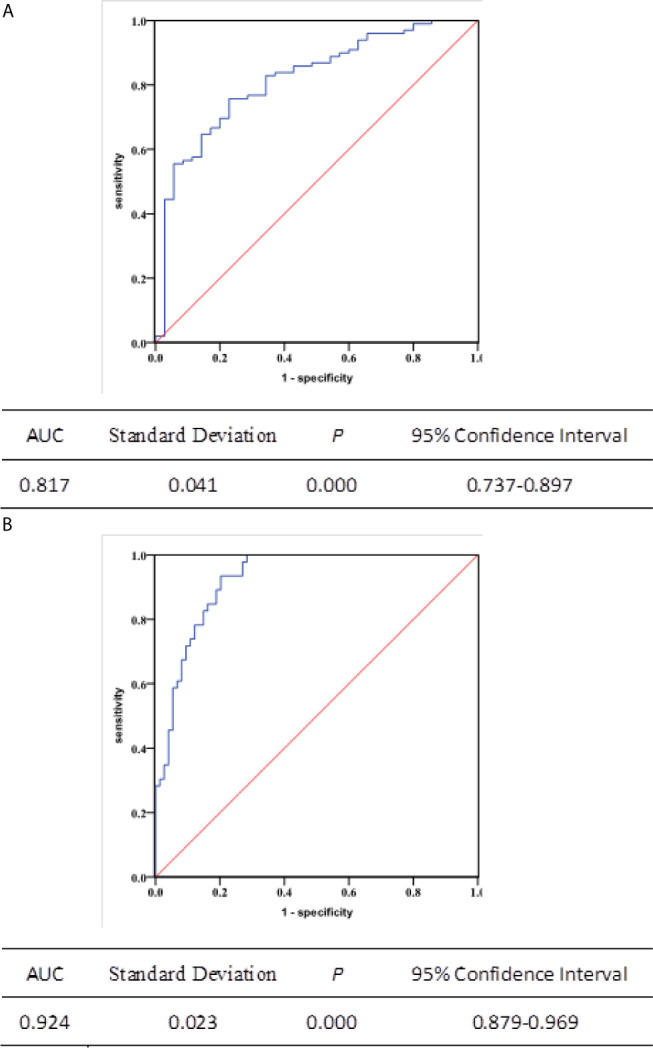
Receiver operating characteristic (ROC) curves depicting the predicting capability of the change of TBF in 134 primary tumors and 120 metastatic lymph nodes. The area under the ROC curve was **(A)** 0.817 (95% confidence interval: 0.737-0.897, *P* < 0.001) and **(B)** 0.924 (95% confidence interval: 0.879-0.969, *P* < 0.001), respectively.

Multivariate logistic regression showed that the change of TBF (HR = 1.072; *P* < 0.001), T-stage (T1+2 *vs.* T3+4: HR = 0.319; *P* = 0.032) and pathological types (keratinizing squamous *vs.* non-keratinizing: HR = 15.619; *P* = 0.015) were independent predictors of primary tumor response to radiotherapy (**Table 2**). We also found that the change of TBF and clinical stage was associated with radiotherapy response of metastatic lymph nodes (HR = 1.231; *P* < 0.001. I+II *vs.* III+IV: HR = 0.084; *P* = 0.009. **Table 3**). In addition, ROC curves were calculated to further evaluate the effectiveness of TBF metrics in discerning CR from PR to radiotherapy. The accuracy of predicting the response for primary tumors and metastatic lymph nodes using percentage change of TBF was 0.817 and 0.924, respectively (*P* < 0.001, *P* < 0.001; **Figure 4**).

**Table 2:** Clinicopathologic characteristics associated with radiotherapy response of primary tumors.

Variable	Univariate		Multivariate	
	HR (95%CI)	*P*-value	HR (95%CI)	*P*-value
Gender				
Male	Reference			
Female	0.673 (0.280-1.619)	0.377		
Age (years)				
≤50	Reference			
>50	1.475 (0.678-3.209)	0.327		
Pathological type				
Keratinizing squamous	Reference		Reference	
Non-keratinizing	3.958 (0.998-15.693)	0.050	15.619 (1.699-143.586)	0.015
Tumor stage				
T1+2	Reference		Reference	
T3+4	0.298 (0.133-0.670)	0.003	0.319 (0.112-0.908)	0.032
Clinical stage				
I+II	Reference		Reference	
III+IV	0.200 (0.045-0.899)	0.036	0.308 (0.037-2.539)	0.274
Therapy				
RT alone	Reference			
Concurrent CRT	0.456 (0.053-3.926)	0.475		
% change of TBF (primary tumor)	1.067 (1.040-1.096)	<0.001	1.702 (1.041-1.103)	<0.001

TBF, tumor blood flow.

**Table 3:** Clinicopathologic characteristics associated with radiotherapy response of metastatic lymph nodes.

Variable	Univariate		Multivariate	
	HR (95%CI)	*P*-value	HR (95%CI)	*P*-value
Gender				
Male	Reference			
Female	1.428 (0.612-3.333)	0.410		
Age (years)				
≤50	Reference			
>50	1.327 (0.634-2.775)	0.453		
Pathological type				
Keratinizing squamous	Reference			
Non-keratinizing	0.930 (0.149-5.785)	0.938		
Tumor stage				
T1+2	Reference			
T3+4	0.729 (0.348-1.520)	0.402		
Clinical stage				
I+II	Reference		Reference	
III+IV	0.100 (0.021-0.481)	0.004	0.084 (0.014-0.542)	0.009
Therapy				
RT alone	Reference			
Concurrent CRT	1.250 (0.110-14.187)	0.857		
% change of TBF (LNM)	1.223 (1.138-1.314)	<0.001	1.231 (1.139-1.331)	<0.001

TBF, tumor blood flow; LNM, lymph node metastasis.

## Discussion

In this study, we demonstrated that TBF is a useful metric to distinguish complete response patients from partial response patients. The conclusions from this work contribute to the development of individualized treatment for patients. Although the clinical use of the TBF parameter in head and neck carcinoma has been recently reported (20), none of the previous works studied NPC patients. To the best of our knowledge, this is the first study that evaluates the predictive accuracy of TBF calculated by pCASL in NPC patients.

Intensity-modulated radiotherapy (IMRT) can provide a good coverage of target volume and reduce exposure to the adjacent normal tissues, which improved the locoregional control rate to 80% - 90% in NPC (21). The low local control rate (73.88%) in our study was due to immediate evaluation after radiotherapy. Some lesions will gradually degrade within three months after radiotherapy because of delayed effect. Our study demonstrated that T-stage is the main influence factor for local control of primary tumors. In previous studies (22), T-stage and primary tumor volume were proved to have a significant impact on the prognosis of NPC patients. Au et al. (23) studied 3328 NPC patients treated with IMRT. They found that the 5-year local control rate for T_1-3_ exceeded 90%, but the local control rate of T_4_ was 71.6%. This is because T_4_ tumors are often close to adjacent critical neurological structures, which compromises the dose of radiation covering the tumor volume and therefore weakens local control. Our findings are consistent with these previous studies.

Our results also demonstrated no direct correlation between SUVmax derived from PET-CT and TBF calculated by ASL in NPC. However, Bisdas et al. showed a positive correlation between SUVmax and TBF (24). The possible explanation is that neovascularity and angiogenesis have been known to develop with proliferation of tumor cells in the early period, and the glucose uptake of a tumor rises in parallel. Komar G previously concluded that there was no correlation between SUV and TBF value in HNSCC patients (25), our study came to a similar conclusion. Fujima (26) showed significant correlations between SUV and TBF in HNSCC by different T-stage and tumor locations, i.e., positive correlation in the early T-stage pharynx/oral carcinoma, negative correlation in the advanced T-stage in both the pharynx/oral and sinonasal carcinoma, and no correlation in the overall patient analysis. The conflicting result may be due to several factors such as tumor size, tumor proliferation activity, or the mismatch of angiogenesis and tumor proliferation. Insufficient blood supply leads to relatively low TBF, whereas an aggressive tumor itself demands a high glucose uptake in anaerobic glycolysis. The uncoupling of blood supply and tumor growth may result in low oxygenation of tumor tissue, i.e., tumor hypoxia, which might lead to resistance to radiotherapy (26). Our study showed no correlation between the SUVmax of primary tumors or metastasis lymph nodes and radiation response. It is possible that tumors with high SUVmax may contain more hypoxic cells, which reduce radiotherapy sensitivity. Moreover, necrosis and inflammatory tumors can raise SUVmax and lower radiotherapy efficacy (27). Further analysis is required to investigate and reveal tumor biological correlations.

The TBF obtained from ASL can assess the response of radiotherapy in NPC patients by a noninvasive manner. We compared the change of TBF and tumor response in primary tumors and metastatic lymph nodes. We observed that the accuracy of TBF for predicting the response of primary tumors or metastatic lymph nodes was 0.817 (*P* < 0.001) and 0.924 (*P* < 0.001). Although tumor response can be directly observed in MRI, it detected the morphologic changes of tumors; and it failed to provide functional information. TBF reflects tumor perfusion, which is closely related to tumor growth. The timely change of TBF can detect early treatment response and improve patient care by tailoring treatment and the management of follow-up. Our study explored that the change of TBF can be used as a valuable biomarker to predict the sensitivity of radiotherapy and suggested that the TBF values or the change of TBF is positive corrected with survival time. In general, the prognosis of patients with PR is worse than patients with CR. Thus, distinguishing patients who will have a partial response to current therapy from patients who will have a complete response to therapy will help clinicians determine the optimal therapy strategy for these patients. King’s study demonstrated that the pre-treatment TBF calculated by a dynamic contrast-enhanced (DCE) perfusion technique can estimate the prognosis of patients and that lower pre-treatment TBF values showed the worse prognosis (28). Fujima N reported TBF (121.4mL/min/100g) reduction after treatment (24.9 mL/min/100 g) in head and neck tumors; and the TBF reduction rate was remarkably lower in complete response patients than without. Previous ASL-related research mostly focused on the central nervous system and clinical usage in tumor diagnosis, specialization, therapeutic effect monitoring, and assessment of prognosis (29). Some other studies concluded that perfusion images from DCE-MRI may support diagnosis and estimating therapeutic outcome, distinguishing tumor recurrence from therapeutic alteration, and predicting prognosis in NPC (30, 31). Lin M reported that TBF obtained from ASL showed good consistency with the parameters of DCE-MRI. They also demonstrated that different perfusion areas in the whole tumor showed a significant correlation coefficient between ASL and DCE-MRI, and thus ASL may be able to provide a reliable perfusion property without invasion and replace DCE-MRI in NPC (32). Higher TBF causes abundant oxygen in tumors, which improves the sensitivity of radiotherapy in NPC, the lower perfusion in heterogeneous tumor areas or necrotic hypoxic lesions may lead to resistance of radiotherapy (33). The large change of TBF with a larger volume reduction may be caused by shrinking the intratumoral arteriovenous shunt or decreasing the vascular chemoradiotherapy. Furthermore, TBF obtained by ASL can be carried out safely and repeatedly at any time of radiotherapy without a contrast agent and radiation exposure. Monitoring of local TBF change may be used to design chemoradiation de-escalation trials to readjust treatment intensity, which is achieved by FDG-PET (34). The timely evaluation of TBF change can provide helpful information for guiding the choice of adjuvant chemotherapy or earlier salvage surgery after radiotherapy. The advanced diffusion parameter of functional MRI converts medical images into quantitative perfusion predictors to provide prognostic ability without increasing economic cost and invasion in NPC. This is a promising area that requires further investigation.

There are several limitations in this study: First, the study sample is a single-center dataset. Therefore, multi-center clinical experiments and large sample sizes are needed for a greater quantity and higher level of evidence to confirm the results of this study. An external validation is needed to validate the effectiveness of the findings. Second, because of the low spatial resolution of the TBF map, we needed to fuse TBF images to T2WI or enhanced T1WI MRI to distinguish anatomical details and outline the ROI. We tested the mean TBF value from ASL by redrawing ROI and did not evaluate the impact of inter-observer variations. Moreover, although this study suggests that the TBF is a promising parameter for evaluating the response of radiation therapy. We failed to find another “gold standard” criterion, which could be used to perform the ROC curves of RECIST; therefore, we could not compare if TBF was as good as RECIST in evaluating the tumor response. Third, different perfusion parameters may have a complex relationship with each other. A significant correlation was confirmed between TBF derived from ASL and parameters of DCE - MRI. Further studies are needed to explore the combination of both perfusion parameters. Fourth, the follow-up time of this study was limited, and no long-term treatment response and overall survival have been investigated yet. Future studies will extend the follow-up time and discuss in detail the relationship between TBF and radiation prognosis. This research project is still ongoing, and related data such as tumor-free survival, overall survival, local control rate, relapse rate, and metastasis rate are being followed up.

## Conclusions

In conclusion, TBF of ASL is a promising metric in evaluating tumor perfusion quantitatively and the change of TBF is a non-invasive choice for accurately predicting response of radiotherapy in NPC. Thus, this study suggests that when patients undergo radiotherapy at 50 Gy, the change of TBF might be a promising parameter which could evaluate the effectiveness of the therapy strategy. This will help clinicians modify the strategy in time and give rise to benefit to patients.

## Data Availability Statement

The original contributions presented in the study are included in the article/supplementary material. Further inquiries can be directed to the corresponding authors.

## Ethics Statement

The studies involving human participants were reviewed and approved by the ethics committee of Shandong Cancer Hospital Affiliated to Shandong University. The patients/participants provided their written informed consent to participate in this study. Written informed consent was obtained from the individual(s) for the publication of any potentially identifiable images or data included in this article.

## Author Contributions

XC and CG designed the experiment, analyzed the experimental raw data, and was a major contributor in writing the manuscript. YS, LW, XY, XB and MH executed the experiment process, recorded the data and revised the manuscript for important intellectual contents. JS and JX checked the experimental raw data. YY made substantial contributions to conception and design the whole experiment. All authors contributed to the article and approved the submitted version.

## Funding

This study was supported by the National Natural Science Foundation of China (Grant No. 82072094), Natural Science Foundation of Shandong Province (Grant Nos. ZR2019LZL017 and ZR2020MH227).

## Conflict of Interest

The authors declare that the research was conducted in the absence of any commercial or financial relationships that could be construed as a potential conflict of interest.

## Publisher’s Note

All claims expressed in this article are solely those of the authors and do not necessarily represent those of their affiliated organizations, or those of the publisher, the editors and the reviewers. Any product that may be evaluated in this article, or claim that may be made by its manufacturer, is not guaranteed or endorsed by the publisher.
